# Comparative study on the predictive value of TG/HDL-C, TyG and TyG-BMI indices for 5-year mortality in critically ill patients with chronic heart failure: a retrospective study

**DOI:** 10.1186/s12933-024-02308-w

**Published:** 2024-06-20

**Authors:** Zijing Zhou, Qiang Liu, Min Zheng, Zhihong Zuo, Guogang Zhang, Ruizheng Shi, Ting Wu

**Affiliations:** 1 Department of Cardiovascular Medicine, National Clinical Research Center for Geriatric Disorders, Xiangya Hospital, Central South University, #87 Xiangya Road, Kaifu District, Changsha, 410008 Hunan China; 2 Department of Gastroenterology and Endoscopy, Eastern Hepatobiliary Surgery Hospital, Naval Military Medical University, Shanghai, China; 3 Department of Critical Care Medicine, Xiangya Hospital, Central South University, Changsha, Hunan China

**Keywords:** Triglyceride glucose index, Triglyceride glucose-body mass index, Triglyceride-density lipoprotein cholesterol ratio, 5-Year mortality, Chronic heart failure

## Abstract

**Background:**

The triglyceride glucose (TyG) index, TyG-body mass index (TyG-BMI), and triglyceride-density lipoprotein cholesterol ratio (TG/HDL-C) are substitute indicators for insulin resistance (IR). This study aimed to compare the predictive value of these indicators for 5-year mortality in critically ill patients with chronic heart failure (CHF).

**Methods:**

Critically ill patients with CHF were identified from the Multiparameter Intelligent Monitoring in Intensive Care (MIMIC) III and IV databases. The primary outcome was 5-year mortality. The relationship between the three indices and mortality risk was determined using multivariate Cox proportional hazards models, Kaplan–Meier (K‒M) analysis and restricted cubic splines analysis. A receiver operating characteristic (ROC) curve was generated to compare the ability of the three indices to predict mortality. Finally, whether the IR indices would further increase the predictive ability of the basic model including baseline variables with a significance level between survivors and non-survivors was evaluated by ROC curve.

**Results:**

Altogether, 1329 patients with CHF were identified from the databases. Cox proportional hazards models indicated that the TyG index was independently associated with an elevated risk of 5-year mortality (hazard ratio [HR], 1.56; 95% confidence interval [CI] 1.29–1.9), while the TyG-BMI index and TG/HDL-C level were significantly associated with 5-year mortality, with an HR (95% CI) of 1.002 (1.000–1.003) and 1.01 (1.00–1.03), respectively. The K–M analysis revealed that the cumulative incidence of all-cause 5-year death increased with increasing quartiles of the TyG index, TyG-BMI index, or TG/HDL-C ratio. According to the ROC curve, the TyG index outperformed the TyG-BMI and TG/HDL-C ratio at predicting all-cause 5-year mortality (0.608 [0.571–0.645] vs. 0.558 [0.522–0.594] vs. 0.561 [0.524–0.598]). The effect of the TyG index on all-cause mortality was consistent across subgroups, with no significant interaction with randomized factors. Furthermore, adding the TyG index to the basic model for 5-year mortality improved its predictive ability (area under the curve, 0.762 for the basic model vs. 0.769 for the basic model + TyG index); however, the difference was not statistically significant.

**Conclusion:**

As continuous variables, all three indices were significantly associated with 5-year mortality risk in critically ill patients with CHF. Although these IR indices did not improve the predictive power of the basic model in patients with CHF, the TyG index appears to be the most promising index (vs. TyG-BMI and TG/HDL-C ratio) for prevention and risk stratification in critically ill patients with CHF.

**Supplementary Information:**

The online version contains supplementary material available at 10.1186/s12933-024-02308-w.

## Introduction

Chronic heart failure (CHF), a leading cause of morbidity and mortality (5-year mortality rate, approximately 50%), impacts more than 64.3 million people worldwide. In addition, patients with CHF have a poor prognosis, especially those hospitalized in the intensive care unit (ICU) [1–3]. The incidence of CHF is estimated to continue increasing, leading to increasing economic and social burdens [4–6]. Therefore, identifying effective risk factors associated with adverse outcomes in patients with CHF is essential to improving patient care and prognosis.

HF, the end stage of various cardiovascular diseases, is a complex clinical syndrome characterized by multiple complex mechanisms [7]. Insulin resistance (IR), a crucial component of metabolic syndrome, refers to a reduced responsiveness of insulin on effector organs or tissues [8]. Several studies have demonstrated a close relationship between IR and the HF development. IR is closely associated with the inactivation of nitric oxide, which is a protective factor for the vascular endothelium, resulting in inappropriate activation of the renin–angiotensin system and systemic low-grade inflammation, thereby exacerbating HF progression [9, 10]. Additionally, IR can reduce glucose bioavailability and induce changes in substrate metabolism, resulting in increased consumption of myocardial oxygen and a reduced compensatory capacity of the myocardium [11–13]. Furthermore, glycolipid metabolism disorders trigger an increase in the production of reactive oxygen species, resulting in mitochondrial dysfunction, endoplasmic reticulum stress, and impaired cardiac calcium signaling [14–16]. Finally, HF can induce IR, resulting in the formation of a vicious cycle between HF and IR, which may also exacerbate the deterioration of cardiac function [17]. Conventional approaches for detecting IR include the hyperinsulinemic–euglycemic clamp technique. However, this approach is difficult to implement in practical clinical settings because of its high cost and time-consuming nature [18]. Fortunately, it is well known that IR is closely associated with insulin-mediated blood glucose disorders, abnormal lipid accumulation, and obesity [19]. Therefore, in previous epidemiological studies, non-insulin-based fasting IR indicators, known as surrogates, were used to identify IR levels.

The triglyceride-to-high-density lipoprotein cholesterol (TG/HDL-C) ratio and triglyceride and glucose index (TyG) were proposed to be useful biomarkers for IR identification because of their significant correlation with hyperinsulinemic–euglycemic clamp results. In addition, these methods are more suitable for clinical practice [20, 21]. Recent research suggests that combining the TyG index with body mass index (TyG-BMI) significantly improves its effectiveness in evaluating IR [22]. Several large-scale studies have recognized TyG index and TG/HDL-C ratio as important factors for predicting cardiovascular disease (CVD) occurrence [23–25]. Recently, the TyG index was identified as a reliable and significant prognostic indicator in patients with HF [26]. Moreover, the TyG-BMI was employed to predict 1-year all-cause mortality in patients with HF [27].

However, each IR index represents a different aspects of insulin resistance. TG are lipids or fats produced by the body to store energy. HDL-C is a beneficial cholesterol molecule that takes up and returns surplus cholesterol from the body to the liver for excretion or re-utilization [28]. Therefore, the TG/HDL-C ratio reflects the lipid metabolism in IR. The TyG index is calculated using fasting TG and glucose levels and reflects the interplay between lipid and glucose metabolisms [29]. TyG-BMI, a product of the TyG index and BMI, further reflects obesity. Therefore, we believe that the predictive abilities of these surrogates differ. Although these indicators have been examined in different cohorts, a head-to-head comparison of their effectiveness at predicting clinical outcomes in patients with HF is lacking. Therefore, this study aimed to compare the ability of these parameters to predict short- and long-term mortality in patients with CHF to provide clinicians with a better predictive tool.

## Method

This retrospective cohort study was performed according to the Strengthening the Reporting of OBservational Studies in Epidemiology (STROBE) guidelines. The project was approved by the Medical Ethics Committee for Clinical Research, Xiangya Hospital, Central South University. All procedures were performed in accordance with the ethical standards of the responsible committee on human experimentation (institutional or regional) and with the Declaration of Helsinki 1975.

### Source of data

The Multiparameter Intelligent Monitoring in Intensive Care III subset and IV (MIMIC III and IV) databases, which are large US-based critical care databases, were applied in the present study. The MIMIC III subset database comprises medical information of patients admitted to the intensive care unit (ICU) of Beth Israel Deaconess Medical Center (BIDMC) between 2001 and 2008, while the MIMIC IV database contains information on ICU patients from the same center between 2008 and 2019. Therefore, the present retrospective study analyzed patients from the BIDMC between 2001 and 2019. The author (TW) was able to access the database and was responsible for the data extraction (Certification Number 41115067).

### Subject selection

Patients who were diagnosed with CHF were included in the present study according to the International Classification of Diseases, Ninth and Tenth Revision (ICD-9 and − 10) codes. The exclusion criteria were as follows: (1) aged < 18 years; (2) admitted to the ICU for < 24 h; (3) missing data on weight, height, TG, HDL, fasting blood glucose and N-terminal pro-brain natriuretic peptide (NT-proBNP) levels; and (4) had multiple admissions, for whom only the first-time data were extracted.

### Variable extraction

The software PostgresSQL (v13.7.1) and Navicate Premium (version 15) were used to extract the data. This set of baseline variables was chosen on the bases of their possible influence on cardiovascular risk of the individuals. The following variable data were extracted: age, sex, weight, height and blood pressure. Patient comorbidities, including hypertension, diabetes, atrial fibrillation (AF), acute myocardial infarction (AMI), chronic kidney disease (CKD), dyslipidemia, prior AMI and respiratory failure, were also collected for analysis. Laboratory variables, including alanine aminotransferase (ALT), aspartate aminotransferase (AST), creatine kinase (CK), CK-MB, serum creatinine (Scr), blood urea nitrogen (BUN), glucose, potassium, calcium, magnesium, sodium, white blood cell (WBC), red blood cell (RBC), hemoglobin (Hb), platelet (PLT) count, NT-proBNP, troponin I, and lipid profiles, were recorded. For patients with multiple measurements, the highest daily value was included in the analysis. Prescriptions, including statins, antiplatelet drugs, anticoagulant drugs, angiotensin-converting enzyme inhibitors/angiotensin receptor blockers (ACEIs/ARBs), β-blockers, and diuretics, were analyzed within 48 h after hospital admission. The value of the TyG index was calculated as ln [fasting TG (mg/dl) × FBG (mg/dl)]/2, while the TyG-BMI was calculated as TyG × BMI.

### Outcomes

The start date of follow-up was the date of hospital admission. The primary outcome was 5-year mortality. The secondary outcomes were hospital mortality and 360-day mortality.

### Statistical analysis

Continuous variables were presented as the mean ± standard deviation or median and interquartile range (IQR), whereas categorical variables were expressed as the total number and frequency. For categorical variables, the X^2^ test or Fisher’s exact test was performed for analysis. Student’s t test and the Wilcoxon rank-sum test were used for continuous variables. Variables with more than 10% missing values were excluded. Variables with missing values between 5% and 10% were processed using multiple interpolation to fill in the missing values with the most suitable set of data. Variables with less than 5% missing values were replaced with the mean of that variable. Variables with abnormal values were addressed using the winsorize method with 1% and 99% as cutoff points. The relationships between the TG/HDL-C ratio, TyG index, and TyG-BMI and the risk of mortality were determined using Kaplan–Meier (K–M) curves, and Cox proportional hazards models. Baseline variables with a significance level of *p* < 0.05 between survivors and non-survivors were included in a multivariate model. In addition, multicollinearity was examined using Variance Inflation Factor (VIF) to ensure variable independence in the study. A recommended maximum VIF value of 5 was used in the study, as suggested by previous studies [30, 31]. In Model 1, adjustments were made for age, AF, diabetes status, CKD status, hypertension status, and respiratory failure. In Model 2, further adjustments were made for laboratory results and prescriptions. Subgroup analysis was conducted to explore the association between continuous the TyG index and 5-year mortality in different subgroups. Restricted cubic splines (RCSs) analysis was used to further investigate the dose‒response relationships between the three indices and mortality. Receiver operating characteristic (ROC) curve analysis was then performed to compare the predictive ability, sensitivity, and specificity of the three indices for assessing mortality. Continuous variables were divided into dichotomous groups based on clinical significance. A *p*-value < 0.05 was considered statistically significant. Stata 12 (STATA Corporation, College Station, Texas, USA) was used to analyze all the data.

## Results

### Baseline characteristics

**Fig. 1:**
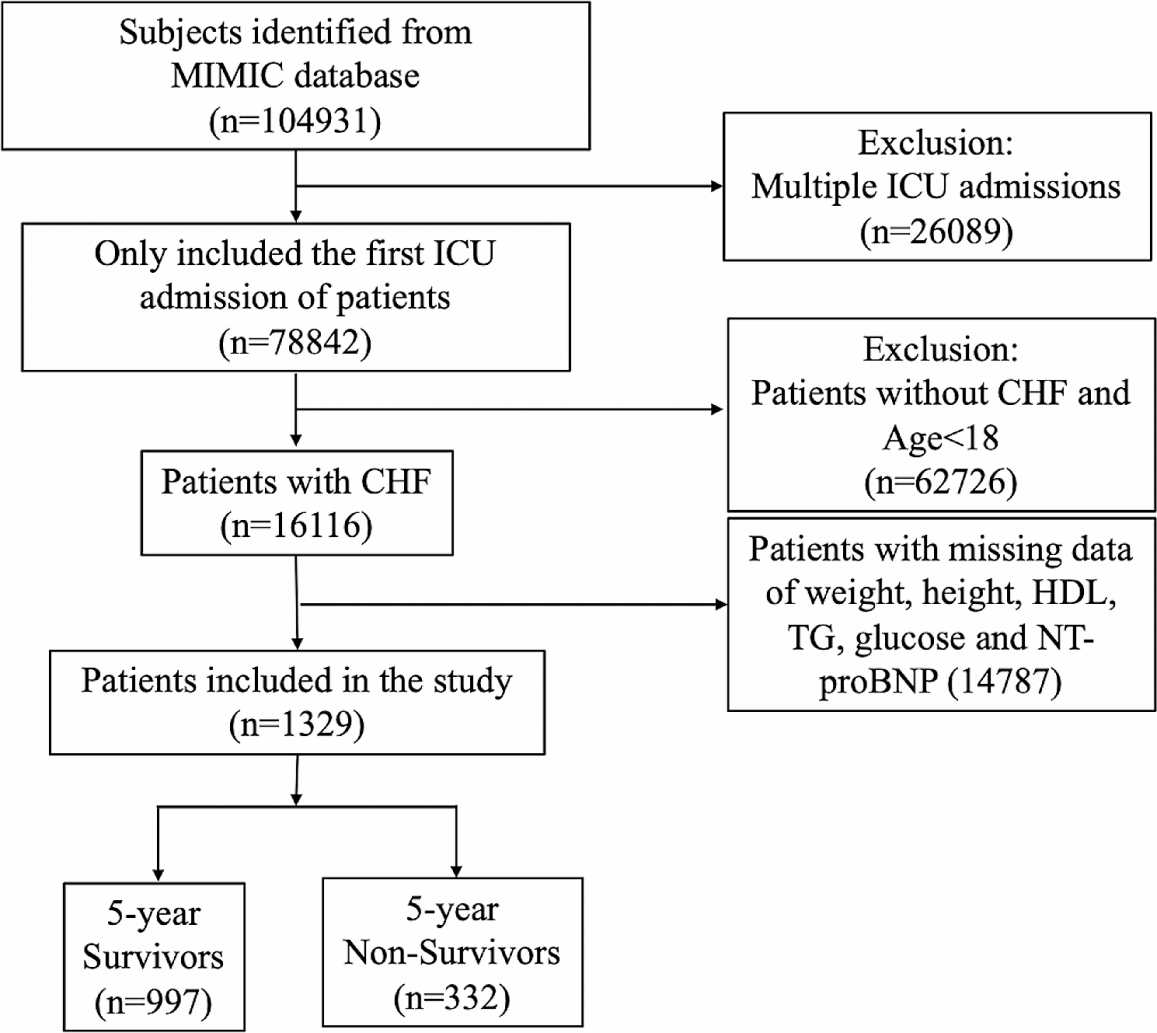
Patient selection process

After screening the data of patients with CHF in the MIMIC III subset and the MIMIC IV database, 1329 patients who fulfilled the inclusion criteria were included in this study. Figure 1 shows the patient selection process. The baseline characteristics of the included patients were categorized based on their 5-year mortality. The 1329 patients were divided into two groups: surviving (997, 75%) and non-surviving (332, 25%). Non-survivors tended to be older than the survivors. In addition, patients with AF, diabetes, CKD and respiratory failure had a higher risk of 5-year mortality. Non-survivors demonstrated significantly higher levels of ALT, AST, RBC, CK, Scr, glucose, HbA1C, NT-proBNP, potassium, sodium, BUN, WBC, and TG. The proportion of patients who received ACEIs/ARBs, antiplatelet drugs, or β-receptor within 48 h was significantly lower among the non-survivors compared to the survivors. Table 1 demonstrates a detailed comparison between the survivors and non-survivors.

**Table 1:** Baseline characteristics between survivors and non-survivors

Variables	Total (*n* = 1329)	Survivor (*n* = 997)	Non-survivor (*n* = 332)	*p*
Gender, N (%)				0.417
0	587 (44.2)	434 (43.5)	153 (46.1)	
1	742 (55.8)	563 (56.5)	179 (53.9)	
Age (years)	71.2 ± 12.9	70.4 ± 13.3	73.4 ± 11.6	**< 0.001**
Height (cm)	167.6 ± 10.8	167.6 ± 11.0	167.5 ± 10.4	0.910
Weight (kg)	79.0 ± 20.2	79.8 ± 20.2	76.6 ± 19.9	**0.012**
BMI	28.5 ± 6.5	28.4 ± 6.6	28.7 ± 6.2	0.516
AF, N (%)				**0.028**
0	654 (49.2)	508 (51)	146 (44)	
1	675 (50.8)	489 (49)	186 (56)	
AMI, N (%)				0.726
0	1133 (85.3)	848 (85.1)	285 (85.8)	
1	196 (14.7)	149 (14.9)	47 (14.2)	
Diabetes, N (%)				**< 0.001**
0	976 (73.4)	758 (76)	218 (65.7)	
1	353 (26.6)	239 (24)	114 (34.3)	
CKD, N (%)				**< 0.001**
0	803 (60.4)	639 (64.1)	164 (49.4)	
1	526 (39.6)	358 (35.9)	168 (50.6)	
Dyslipidemia, N (%)				0.071
0	748 (56.3)	547 (54.9)	201 (60.5)	
1	581 (43.7)	450 (45.1)	131 (39.5)	
Hypertension, N (%)				**< 0.001**
0	832 (62.6)	599 (60.1)	233 (70.2)	
1	497 (37.4)	398 (39.9)	99 (29.8)	
Old-AMI, N (%)				0.923
0	1135 (85.4)	852 (85.5)	283 (85.2)	
1	194 (14.6)	145 (14.5)	49 (14.8)	
Respiratory failure, N (%)				**< 0.001**
0	892 (67.1)	721 (72.3)	171 (51.5)	
1	437 (32.9)	276 (27.7)	161 (48.5)	
ALT, (IU/L)	215.9 ± 506.0	186.3 ± 463.4	304.7 ± 608.7	**< 0.001**
AST, (IU/L)	353.4 ± 956.4	283.0 ± 824.3	564.7 ± 1251.2	**< 0.001**
Calcium, (mg/dl)	9.3 ± 1.0	9.3 ± 1.0	9.3 ± 0.9	0.371
RBC, (K/UL)	4.0 ± 0.7	4.1 ± 0.7	3.9 ± 0.6	**< 0.001**
CK, (U/L)	698.3 ± 1749.6	632.9 ± 1551.8	894.6 ± 2232.7	**0.018**
CK-MB, (IU/L)	29.4 ± 66.1	27.4 ± 62.0	35.1 ± 77.0	0.066
Scr, (mg/dl)	2.3 ± 1.9	2.1 ± 1.9	2.9 ± 2.0	**< 0.001**
Glucose, (mg/dl)	211.5 ± 116.7	195.4 ± 89.2	259.7 ± 166.3	**< 0.001**
HbA1c, (%)	7.0 ± 1.7	6.9 ± 1.6	7.2 ± 1.7	**0.002**
Hb, (g/dl)	11.9 ± 2.0	12.1 ± 2.0	11.4 ± 1.8	**< 0.001**
Magnesium, (mg/dl)	2.7 ± 1.1	2.7 ± 1.1	2.7 ± 1.0	0.809
PLT, (K/UL)	296.2 ± 143.7	297.1 ± 140.3	293.4 ± 153.6	0.678
NT-proBNP, (pg/ml)	12139.9 ± 14064.0	11038.2 ± 13669.5	15448.0 ± 14720.2	**< 0.001**
Potassium, (mEq/L)	5.2 ± 0.9	5.2 ± 0.9	5.4 ± 1.0	**0.001**
Sodium, (mEq/L)	143.3 ± 5.1	142.9 ± 4.7	144.2 ± 6.1	**< 0.001**
BUN, (mg/dl)	50.1 ± 30.9	44.8 ± 27.4	66.0 ± 35.2	**< 0.001**
WBC, (K/UL)	16.6 ± 10.3	15.7 ± 8.6	19.5 ± 13.9	**< 0.001**
Troponin-I, (ng/ml)	1.3 ± 2.7	1.2 ± 2.7	1.3 ± 2.5	0.611
TG, (mg/dl)	151.0 ± 82.3	146.6 ± 75.8	164.2 ± 98.2	**< 0.001**
LDL, (mg/dl)	97.8 ± 44.7	99.0 ± 44.9	94.2 ± 44.0	0.084
HDL, (mg/dl)	47.9 ± 21.4	48.5 ± 20.9	46.3 ± 22.7	0.117
Cholesterol, (mg/dl)	173.9 ± 56.1	175.1 ± 55.5	170.4 ± 57.5	0.189
PCI, N (%)				0.164
0	1271 (95.6)	949 (95.2)	322 (97)	
1	58 (4.4)	48 (4.8)	10 (3)	
Statin, N (%)				0.109
0	614 (46.2)	448 (44.9)	166 (50)	
1	715 (53.8)	549 (55.1)	166 (50)	
ACEI/ARB, N (%)				**< 0.001**
0	982 (73.9)	704 (70.6)	278 (83.7)	
1	347 (26.1)	293 (29.4)	54 (16.3)	
Anti-platelet drugs, N (%)				**< 0.001**
0	564 (42.4)	394 (39.5)	170 (51.2)	
1	765 (57.6)	603 (60.5)	162 (48.8)	
Anti-coagulant drugs, N (%)				0.079
0	298 (22.4)	212 (21.3)	86 (25.9)	
1	1031 (77.6)	785 (78.7)	246 (74.1)	
β-receptor, N (%)				**< 0.001**
0	486 (36.6)	334 (33.5)	152 (45.8)	
1	843 (63.4)	663 (66.5)	180 (54.2)	
Diuretics, N (%)				0.851
0	462 (34.8)	348 (34.9)	114 (34.3)	
1	867 (65.2)	649 (65.1)	218 (65.7)	
MRA, N (%)				0.715
0	1262 (95)	948 (95.1)	314 (94.6)	
1	67 (5.0)	49 (4.9)	18 (5.4)	
TyG	9.5 ± 0.6	9.4 ± 0.6	9.7 ± 0.8	**< 0.001**
TG/HDL-C	4.2 ± 4.8	3.9 ± 4.5	4.9 ± 5.5	**0.002**
TyG-BMI	270.0 ± 66.6	267.1 ± 66.4	278.6 ± 66.7	**0.006**

Bold values denote statistical significance at the *p* < 0.05 level

ACEI/ARB, angiotensin-converting enzyme inhibitor/angiotonin receptor blocker; AF, atrial fibrillation; AKI, acute kidney injury; ALT, alanine aminotransferase; AMI, acute myocardial infarction; AST, aspartate aminotransferase; BMI, body mass index; BUN, blood urea nitrogen; CK, creatine kinase; CKD, chronic kidney disease; Hb, hemoglobin; HbA1c, hemoglobin A1c; HDL-C, high density lipoprotein cholesterol; LDL-C, Low density lipoprotein cholesterol; NT-proBNP, N-Terminal Pro-Brain Natriuretic Peptide; PCI, percutaneous coronary intervention; PLT, platelet; RBC, red blood cell; Scr, serum creatinine; TG, triglycerides; WBC, white blood cell

### Associations between the TyG index and mortality risk

When considering the TyG index as a continuous variable, Cox proportional hazards analysis revealed a significant association between the risk of 5-year mortality and the TyG index. This association was observed in both the unadjusted model (hazard ratio [HR] 1.95; 95% confidence interval [CI] 1.66–2.29) and the fully adjusted model (HR 1.56; 95% CI 1.29–1.9). The patients were then divided into four groups based on the quartile of the TyG index: T1 (TyG ≤ 9.03, *N* = 333), T2 (TyG > 9.03, ≤ 9.45; *N* = 333), T3 (> 9.45, ≤ 9.85; *N* = 333), and T4 (> 9.85; *N* = 330). The Cox proportional hazards analysis demonstrated that the highest quartile (T4) of the TyG index was significantly associated with the risk of 5-year mortality in both the unadjusted model (HR 2.26; 95% CI 1.66–3.08) and the adjusted model (Model 1: HR 1.96; 95% CI 1.42–2.72; Model 2: HR 1.44; 95% CI 1.01–1.24) (Table 2). We also provided HR and *p* value of all variables in the model 2 in Supplementary Table 3. A similar association was observed between the TyG index and in-hospital mortality and 360-day mortality (Supplementary Tables 1 and 2).

**Table 2:** Association between IR related index and 5-year mortality (Cox regression)

Index	Groups	Non-adjusted HR (95% CI) *P*-Value	Model 1 HR (95% CI) *P*-Value	Model 2 HR (95% CI) *P*-Value
TyG	Continuous	**1.95 (1.66–2.29) < 0.001**	**1.83 (1.53–2.18) < 0.001**	**1.56 (1.29–1.9) < 0.001**
	T1 (≤ 9.03; *N* = 333)	Ref	Ref	Ref
	T2 (> 9.03, ≤ 9.45; *N* = 333)	1.38 (0.99–1.92) 0.058	1.35 (0.97–1.89) 0.076	1.13 (0.80–1.59) 0.476
	T3 (> 9.45, ≤ 9.85; *N* = 333)	1.15 (0.81–1.62) 0.442	1.06 (0.75–1.5) 0.747	0.87 (0.61–1.25) 0.456
	T4 (> 9.85; *N* = 330)	**2.26 (1.66–3.08) < 0.001**	**1.96 (1.42–2.72) < 0.001**	**1.44 (1.01–1.24) 0.042**
	P for trend	**< 0.001**	**< 0.001**	0.083
TyG-BMI	Continuous	**1.002 (1.001–1.003) 0.007**	**1.002 (1.000-1.003) 0.02**	**1.002 (1.000-1.003) 0.04**
	T1 (≤ 225.06; *N* = 332)	Ref	Ref	Ref
	T2 (> 225.06, ≤ 261.65; *N* = 333)	0.92 (0.66–1.29) 0.648	0.93 (0.66–1.3) 0.674	0.89 (0.63–1.26) 0.522
	T3 (> 261.65, ≤ 305.03; *N* = 331)	**1.39 (1.02–1.89) 0.035**	**1.43 (1.04–1.96) 0.027**	1.32 (0.95–1.83) 0.094
	T4 (> 305.03; *N* = 333)	**1.47 (1.08–1.99) 0.014**	**1.44 (1.05–1.29) 0.025**	1.33 (0.95–1.87) 0.100
	P for trend	**0.002**	**0.003**	**0.022**
TG/HDL-C	Continuous	**1.02 (1.01–1.04) 0.003**	**1.02 (1.01–1.04) 0.004**	**1.01 (1.00-1.03) 0.05**
	T1 (≤ 1.85; *N* = 333)	Ref	Ref	Ref
	T2 (> 1.85, ≤ 3.11; *N* = 331)	0.96 (0.69–1.33) 0.806	0.94 (0.67–1.31) 0.704	0.86 (0.61–1.2) 0.364
	T3 (> 3.11, ≤ 4.88; *N* = 334)	1.08 (0.78–1.48) 0.649	1.1 (0.8–1.52) 0.567	1.03 (0.74–1.43) 0.861
	T4 (> 4.88; *N* = 331)	**1.69 (1.26–2.28) 0.001**	**1.69 (1.25–2.28) 0.001**	**1.43 (1.05–1.96) 0.025**
	P for trend	**< 0.0001**	**< 0.001**	**0.007**

Bold values denote statistical significance at the *p* < 0.05 level

Model 1: Age, AF, Diabetes, CKD, Hypertension, Respiratory failure

Model 2: Age, AF, Diabetes, CKD, Hypertension, Respiratory failure, ALT, AST, RBC, CK, Creatinine, HbA1c, Hb, NT-proBNP, potassium, sodium, BUN, WBC, ACEI/ARB, Anti-platelet drugs, β-receptor

**Fig. 2:**
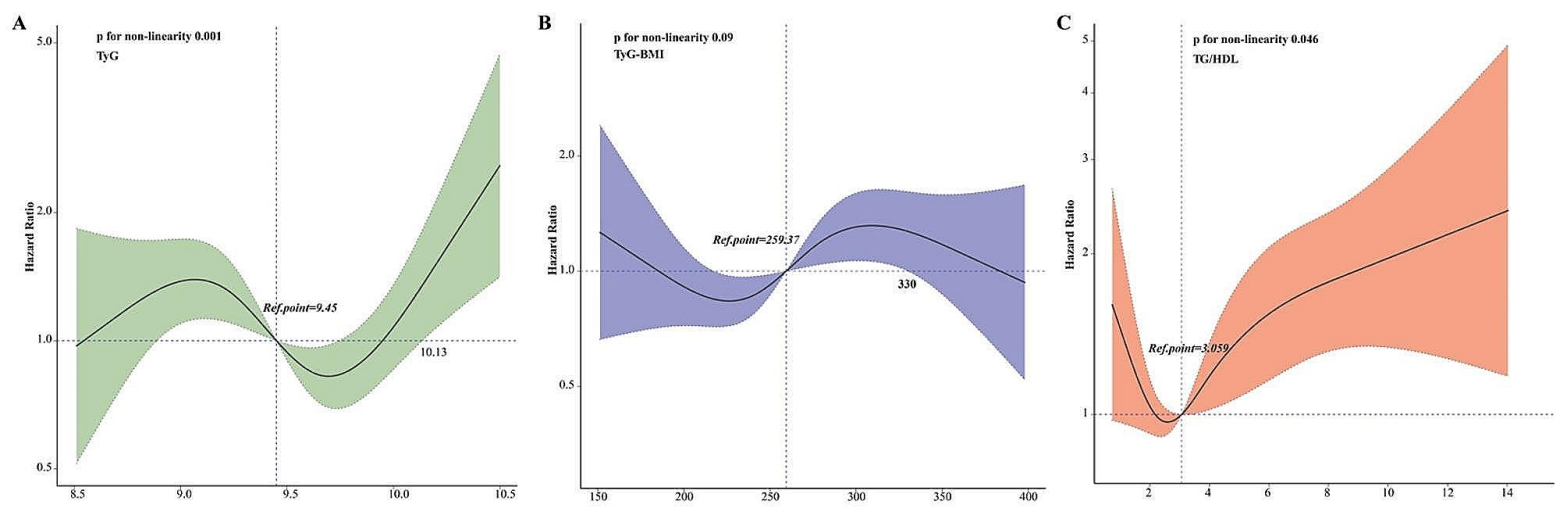
Restricted cubic spline regression analysis of the three indices for 5-year mortality among patients with CHF. **A** TyG index. **B** TyG-BMI index. **C** TG/HDL-C

Notably, we found that *p*-value for trend (0.083) was not significant in adjusted Model 2, which suggested a nonlinear relationship between the TyG index and mortality. Therefore, restricted cubic splines (RCSs) analysis was employed to further investigate this association. The adjusted RCS plots revealed nonlinear associations between the TyG index and 5-year mortality (Fig. 2A, nonlinear *p* = 0.001).

**Fig. 3:**
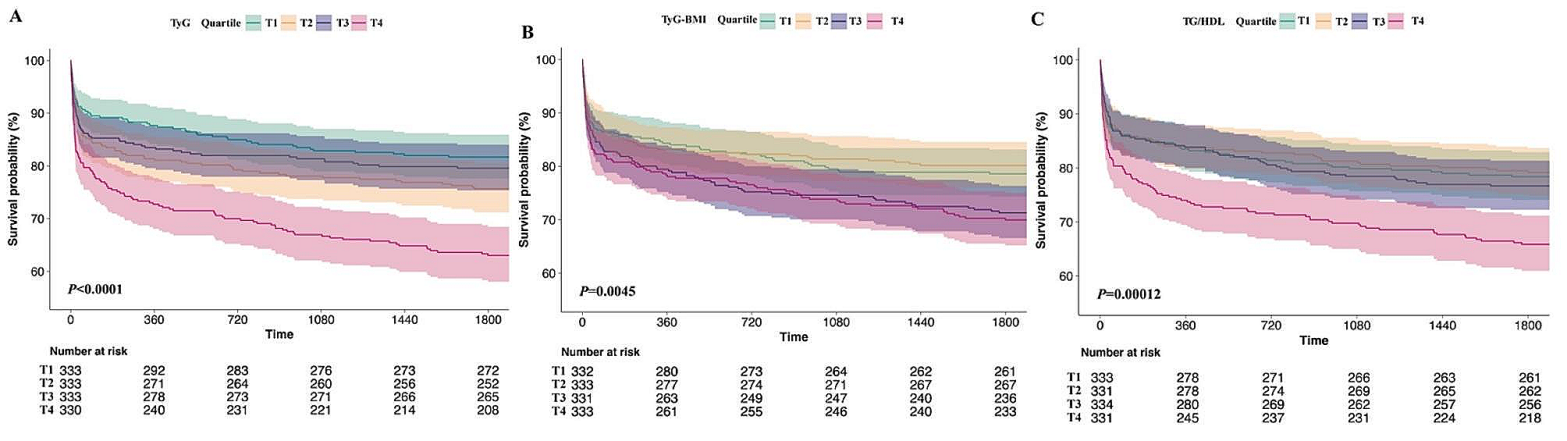
K‒M survival analysis curves for 5-year mortality in patients with CHF. **A** TyG index. **B** TyG-BMI index. **C** TG/HDL-C

During the 5-year follow-up, 332 incident cases of all-cause mortality were reported. The mortality across TyG index groups is illustrated in Fig. 3A (T1: 61, 18.3%; T2: 81, 24.3%; T3: 68, 20.4%; T4: 122, 37.0%), and a significant difference in mortality was observed among these groups (*p* < 0.001).

### Associations between the TyG-BMI and the risk of mortality

Cox proportional risk analysis indicated a significant association between TyG-BMI index and 5-year mortality, in both unadjusted model (HR 1.002; 95% CI 1.001–1.003) and fully adjusted model (HR 1.002; 95% CI 1.000–1.003) when the TyG-BMI index was a continuous variable. When the TyG-BMI index was a nominal variable (quartile T1: ≤225.06; T2: 225.06–261.65; T3: 261.65–305.03; T4: >305.03), it was also associated with a higher incidence of all-cause death within 5 years according to both the unadjusted model (T1 vs. T2: HR 0.92; 95% CI 0.66–1.29; T3: HR 1.39; 95% CI 1.02–1.89; T4: HR 1.47; 95% CI 1.08–1.99; P for trend = 0.002 ) and Model 1 (T1 vs. T2: HR, 0.93; 95% CI 0.66–1.3; T3: HR 1.43; 95% CI 1.04–1.96; T4: HR 1.44; 95% CI 1.05–1.29; P for trend = 0.003) and displayed a tendency to increase with the TyG-BMI index. However, after adjusting for laboratory confounding factors, patients with a higher TyG-BMI tended to suffer from a greater incidence of 5-year mortality; however, the difference was not significant (T1 vs. T2: HR 0.89; 95% CI 0.63–1.26; T3: HR 1.32; 95% CI 0.95–1.83; T4: HR 1.33; 95% CI 0.95–1.87; P for trend = 0.022) (Table 2). We also provided HR and *p* value of all variables in the model 2 in Supplementary Table 4. The influence of the TyG-BMI on hospital and 360-day mortality was also investigated and is presented in Supplementary Tables 1 and 2.

The RCS model revealed a nonlinear connection between TyG-BMI and the all-cause death rate over a 5-year period, with TyG-BMI being kept as a continuous variable (Fig. 2B Nonlinear *P* = 0.09). K‒M curves of the incidence of 5-year mortality for the TyG-BMI quartiles are presented in Fig. 3B. The findings revealed that the cumulative incidence of all-cause 5-year death increased with increasing quartiles of the TyG-BMI (*p* = 0.0045).

### Association between the TG/HDL-C ratio and mortality risk

When the TG/HDL-C index was a continuous variable, it was closely associated with 5-year mortality (unadjusted model: HR 1.02; 95% CI 1.01–1.04; Model 1: HR 1.02; 95% CI 1.01–1.04; Model 2: HR 1.01; 95% CI 1.00–1.03). Patients were divided into four groups according to the quartiles of TG/HDL ratios (quartile T1: ≤1.85; T2: 1.85–3.11; T3: 3.11–4.88; T4: >4.88). After fully adjusting for potential confounders, patients in the highest quartile of the TG/HDL-C ratio had significantly greater risks of 5-year mortality than did those in the lowest quartile, according to Cox proportional risk analysis (T1 vs T2: HR 0.86; 95% CI 0.61–1.2; T3: HR 1.03; 95% CI 0.74–1.43; T4: HR 1.43; 95% CI 1.05–1.96; P for trend = 0.007) (Table 2). We also provided HR and *p* value of all variables in the model 2 in Supplementary Table 5. RCS analysis indicated that the TG/HDL-C ratio was correlated with the risk of 5-year mortality in an inverted V-shaped dose‒response relationship (Fig. 2C, Nonlinear *P* = 0.046). The K‒M survival analysis curve for assessing the incidence of all-cause 5-year mortality among groups based on the quartile groupings of the TG/HDL ratio is shown in Fig. 3C. There was a statistically significant difference in the mortality rate between the groups (T1: 21.6% vs. T2: 20.9% vs. T3: 23.4% vs. T4: 34.1%, *p* = 0.00012).

### ROC curve analysis of TyG, TyG-BMI, and TG/HDL-C

**Fig. 4:**
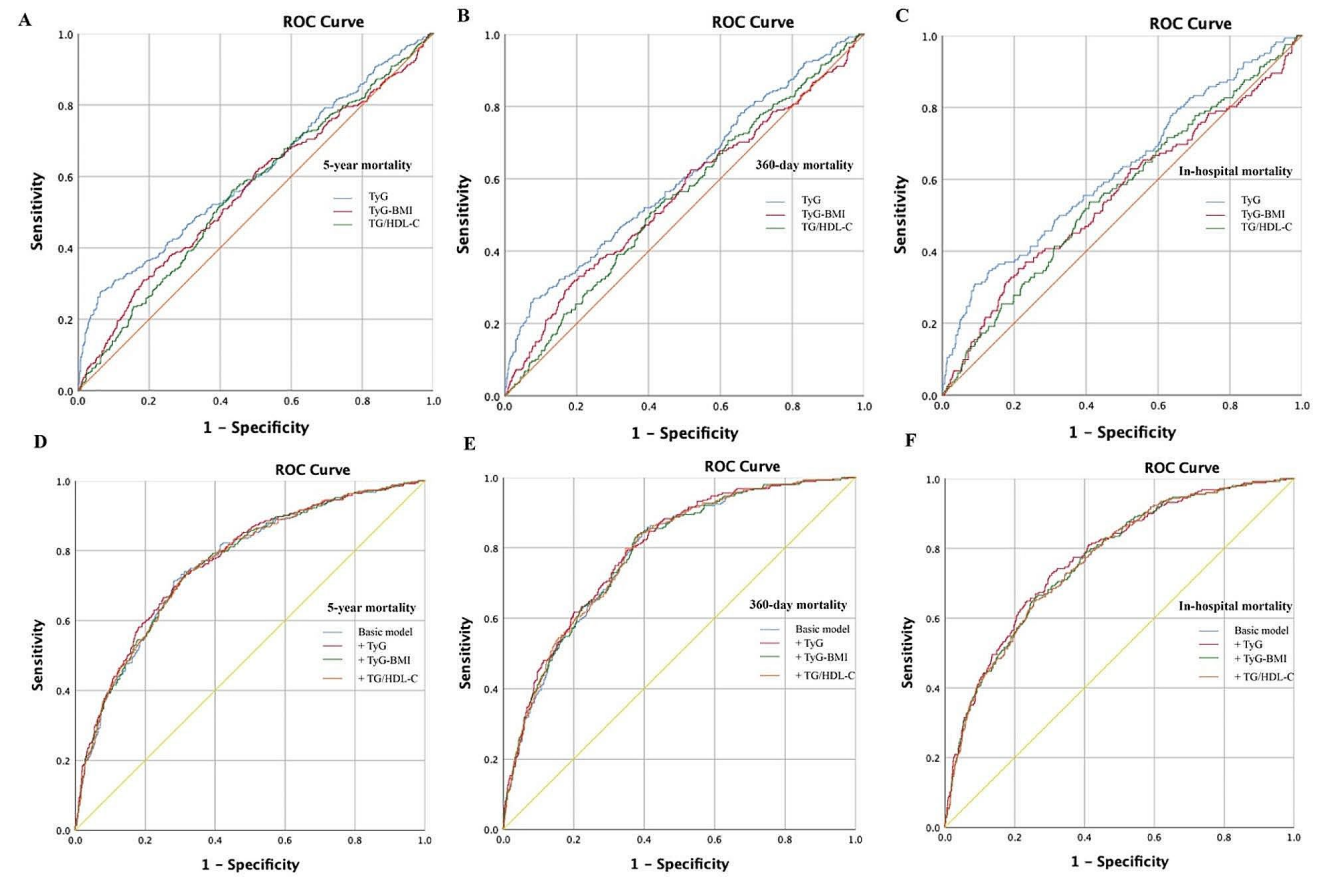
The ROC curves of the IR indices as a marker to predict 5-year, 360-day and hospital mortality. **A** TyG versus TyG-BMI versus TG/HDL-C to predict 5-year mortality. **B** TyG versus TyG-BMI versus TG/HDL-C to predict 360-day mortality. **C** TyG versus TyG-BMI versus TG/HDL-C to predict hospital mortality. **D** Basic risk model versus +TyG, +TyG-BMI, + TG/HDL-C to predict 5-year mortality. **E** Basic risk model versus +TyG, +TyG-BMI, + TG/HDL-C to predict 360-day mortality. **F** Basic risk model versus +TyG, +TyG-BMI, + TG/HDL-C to predict hospital mortality

**Fig. 5:**
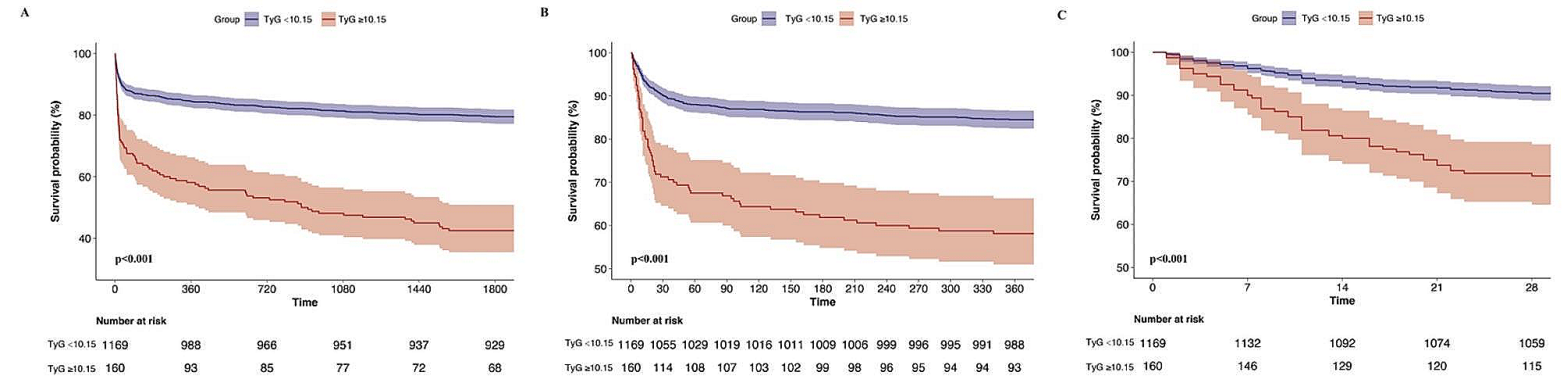
K‒M survival analysis curves for mortality in patients with CHF. **A** 5-Year mortality. **B** 360-Day mortality. **C** Hospital mortality

The ROC curve for the ability of the three indices to predict mortality in patients with CHF is shown in Fig. 4 and Supplementary Table 6. According to the results, the TyG index outperformed the TyG-BMI and TG/HDL ratio in predicting all-cause 5-year mortality [0.608 (0.571–0.645) vs. 0.558 (0.522–0.594) vs. 0.561 (0.524–0.598)], 360-day mortality [0.607 (0.566–0.647) vs. 0.547 (0.508–0.587) vs. 0.556 (0.514–0.597)], and hospital mortality [0.624 (0.575–0.673) vs. 0.559 (0.512–0.607) vs. 0.554 (0.503–0.605)]. The cutoff values for the TyG index for predicting 5-year mortality, 360-day mortality and hospital mortality were 10.15. Based on the results, we divided patients into two groups (≥ 10.15 and < 10.15) and conducted Cox proportional risk analysis and K–M analysis. The results indicated that patients with the TyG index ≥ 10.15 had a significantly higher mortality rate than did those with TyG index < 10.15 (Fig. 5 and Supplementary Table 7). We subsequently conducted subgroup analyses to evaluate the relationship between the TyG index and 5-year mortality in the different subgroups, and no significant interaction was detected (Supplementary Table 8).

Finally, whether the IR indices would further increase the predictive ability of the basic model (including age, AF, Diabetes, CKD, Hypertension, Respiratory failure, ALT, AST, RBC, CK, creatinine, HbA1c, Hb, NT-proBNP, potassium, sodium, BUN, WBC, ACEI/ARB, Antiplatelet drugs, β-receptor). The area under the curve (AUC) and *p* for comparison are presented in Fig. 4 and Table 3. Unfortunately, the results showed no significant incremental predictive ability of the three IR indices to the basic risk model in patients with CHF.

**Table 3:** Improvement in discrimination for mortality after adding IR indices

Models	AUC (95% CI)	*P* value*
5-Year mortality		
Basic model	0.762 (0.733–0.792)	
+TyG index	0.769 (0.740–0.799)	0.167
+TyG-BMI index	0.763 (0.734–0.793)	0.733
+TG/HDL-C	0.763 (0.734–0.792)	0.664
360-Day mortality		
Basic model	0.783 (0.754–0.813)	
+TyG index	0.791 (0.762–0.820)	0.137
+TyG-BMI index	0.784 (0.754–0.814)	0.759
+TG/HDL-C	0.786 (0.756–0.815)	0.125
Hospital mortality		
Basic model	0.764 (0.733–0.796)	
+TyG index	0.773 (0.742–0.805)	0.069
+TyG-BMI index	0.766 (0.734–0.798)	0.462
+TG/HDL-C	0.764 (0.733–0.796)	0.943

* Comparation with basic model

The basic model included Age, AF, Diabetes, CKD, Hypertension, Respiratory failure, ALT, AST, RBC, CK, Creatinine, HbA1c, Hb, NT-proBNP, potassium, sodium, BUN, WBC, ACEI/ARB, Anti-platelet drugs, β-receptor;

## Discussion

To the best of our knowledge, this study was the first to examine the association between the TG/HDL-C ratio and all causes of death in patients with CHF. Furthermore, this study is the first study to investigate the relationship between the TyG-BMI and long-term mortality in patients with CHF. We observed that all the three IR indices as continuous variables were significantly associated with the risk of 5-year mortality in patients with CHF. However, when the TyG-BMI index was considered a nominal variable, patients with a higher TyG-BMI tended to have a higher incidence of 5-year mortality; however, the difference was not significant. The three IR indices did not significantly optimize the predictive performance of basic risk model for risk of 5-year mortality, but the TyG index appears to be the most promising index for prevention and risk stratification in patients with CHF.

In recent years, several clinical studies have shown the correlation between TyG index and the incidence and development of HF. A cohort study including 138,620 participants demonstrated that after adjusting for confounding factors, individuals in the highest quartile of the TyG index exhibited a greater risk of HF than did those in the lowest quartile group. The RCS model revealed a significant J-shaped connection between the TyG index and the incidence of HF [32]. Various analyses have presented similar conclusions [33–35]. Huang et al. evaluated the prognostic value of the TyG index in patients with acute decompensated HF (ADHF) [36]. The results demonstrated that an elevated TyG index could be an independent predictor of adverse events in patients with ADHF. Furthermore, a retrospective cohort study in China, with a median follow-up duration was 3.9 years, included 6697 patients with HF. The results indicated that the TyG index was significantly associated with the risk of mortality, suggesting that the TyG index may be a reliable and valuable predictor for risk stratification and an effective prognostic indicator in patients with HF [26]. This study was the first to investigate the impact of the TyG index on long-term outcomes in patients with CHF. In contrast, we included patients from U.S.-based critical care databases, thus providing more comprehensive results for clinical practice. Moreover, we first reveled the strong correlation between short-term mortality and the TyG index. According to the RCS model, patients in the high TyG index group (≥ 10.13) exhibited a poorer prognosis, as either elevated glucose or elevated lipids can increase the risk of death, reinforcing Zhou’s findings. The increased risk of death in patients with a low TyG index may be attributed to extreme hypoglycemia.

At present, relatively few studies have evaluated the relationship between the TyG-BMI and HF incidence. A recent cross-sectional study confirmed that patients with diabetes or prediabetes with a high TyG-BMI were more likely to have HF. The RCS analysis indicated that a high TyG-BMI had a dose‒response relationship with HF development. The risk is higher when the index exceeds 258.26 [37]. Only one study has elucidated the connection between the TyG-BMI and 1-year all-cause mortality in patients with HF [27]. After adjusting for various confounding factors, a higher TyG-BMI (> 289) was found to be significantly correlated with a lower mortality rate. The “obesity paradox” was used to explain this conclusion. This study is the first to assess the relationship between the TyG-BMI and the long-term prognosis of patients with HF. According to our results, TyG-BMI ≥ 330 was significantly correlated with a lower mortality rate based on the RCS curve, which was consistent with the aforementioned study [27]. The risk of death was the lowest for those with a TyG-BMI in the middle range (216–259.37). Having a higher TyG-BMI tends to increase the risk of mortality, which is understandable as greater adiposity is clearly associated with a poor prognosis in patients with HF [38]. Similarly, the “obesity paradox” may explain why individuals with an extremely high TyG-BMI (> 330) have a greater risk of mortality. Based on our current knowledge, this is the first study to evaluate the effects of TG/HDL-C on the clinical outcomes of patients with HF. Our findings demonstrated that an increased TG/HDL-C ratio was independently associated with 5-year mortality among patients with HF, suggesting that it may serve as an useful marker for the early identification of patients at high risk and with poor outcomes.

Furthermore, we evaluated the performance of the three IR indices in predicting 5-year mortality in patients with CHF. Although the three indices did not improve the predictive power of the basic risk model, the TyG index appears to have a better predictive ability than do the other two indices. Firstly, the positive association of TyG index with mortality remains robust in 5-year, 360-day, and hospital mortality. Furthermore, significant associations were observed irrespective of whether it was treated as a continuous or categorical variable. In addition, the TyG index outperformed the TyG-BMI and TG/HDL-C ratio in predicting all-cause mortality according to ROC (5-year: 0.608 vs. 0.559 vs. 0.561; 360-day: 0.607 vs. 0.547 vs. 0.556; hospital mortality: 0.624 vs. 0.559 vs. 0.554). There are several possible explanations for these results. First, TyG may be a more favorable predictor than the TG/HDL-C ratio because the combination of lipid profiles and FPG may better explain adiposity and cardiometabolic risk factors. Second, the present study’s finding suggest that an elevated baseline TyG index (≥ 10.13) exhibited a poorer prognosis among patients with HF after the adjustment for confounding factors. Meanwhile, the risk of death increases in patients with a low TyG index owing to extreme hypoglycemia. TyG-BMI is an indicator of obesity and a risk factor for cardiovascular diseases. However, many studies reported that patients with HF and a high BMI were more likely to have a better prognosis than those with a low BMI due to “obesity paradox” [27, 39], suggesting the different influence of TyG and BMI trends on prognosis in patients with HF. Combining the TyG index and BMI may make it difficult to achieve better TyG-BMI performance at predicting mortality in patients with HF. To the best of our knowledge, this is the first study to compare the predictive values of TG/HDL-C, TyG, and TyG-BMI for 5-year mortality among patients with CHF using a head-to-head approach in the same population.

Interestingly, a well-organized study by Zhou et al [26] demonstrated that the addition of the TyG index could slightly but significantly improve the AUC obtained from the MAGGIC score in patients with CHF. Adding the TyG index to the basic model for 5-year mortality slightly improved its predictive ability (AUC, 0.762 for the basic model vs. 0.769 for the basic model + TyG index), but this was not statistically significant. There are several explanations for these different conclusions. First, we focused on critically ill patients with CHF, all of whom were admitted to the ICU. This is also one of our advantages. Zhou et al. excluded patients with CKD and severe hepatic impairment, a patient profile that is completely different from that of our study. Interactions among various severe comorbidities in critically ill patients may make it challenging for the TyG index to predict mortality. Second, the MAGGIC score mentioned in a previous study was a simple tool for the prediction of mortality in patients with HF with preserved ejection fraction (HFpEF) and consisted of age, sex, systolic blood pressure, BMI, smoking status, LVEF, NYHA class, creatinine level, HF duration, diabetes status, chronic obstructive pulmonary disorder status, and β‑blockers and ACEI/ARB use [40]. However, > 50% of the participants included in the Zhou study had non-HFpEF. Thus, it is unknown whether the predictive power of the MAGGIC score is suitable for patients with CHF regardless of subtypes. In addition, Zhou ignored other variables that impacted mortality. The basic model in our study included all baseline variables with a significance level of *p* < 0.05 between survivors and non-survivors, and more risk factors were evaluated in the model. Third, the present study included only 1329 patients, which is significantly fewer than the population of the Zhou study. Although the results showed no significant incremental predictive ability of the TyG index in the basic risk model in patients with CHF in the present study, expanding the sample size in future studies may lead to different conclusions. Finally, owing to limited data, we could not calculate the MAGGIC score. However, we attempted to conduct another model that included variables that were similar to those of the MAGGIC score (Supplementary Table 9). Interestingly, we found that adding the TyG index to the model for 5-year mortality significantly improved the AUC, suggesting that the TyG index may be a reliable, valuable, and effective prognostic indicator in patients with CHF. Therefore, establishing a gold standard for predicting the mortality in patients with CHF is necessary. The predictive ability of the TyG index can be accurately evaluated by comparing its results with those of the gold standard.

Patients with diabetes are more likely to develop HF than those without diabetes [41]. Similarly, HF is associated with a higher prevalence of diabetes [41]. In a previous clinical trial, diabetes was associated with a greater risk of death and hospitalization among patients with HF [42]. Therefore, in the present study, we performed a subgroup analysis; its results demonstrated that the TyG index was significantly associated with an increased risk of all-cause mortality after stratification by diabetes status, suggesting that IR affects the prognosis of patients with HF independent of diabetes. These results are consistent with previous conclusions [26, 43, 44]. This phenomenon may be explained as follows. Abnormal glucose regulation is commonly observed in non-diabetic patients with HF [45]. Among patients with HF, those with pre-diabetes or abnormal glucose metabolism were at increased risk of mortality [46].Suskin et al. validated that glucose dysregulation was associated with HF severity in a nondiabetic HF cohort [47]. Therefore, we believe that both diabetic and non-diabetic patients with HF could benefit from the routine use of IR indices index to assess their clinical outcomes. Interestingly, one study reported no significant difference in the risk of worsening HF between the intensive glycemic control and standard treatment arms [48], suggesting that intensive glycemic control alone is insufficient to improve the prognosis of patients with HF. Therefore, recent clinical trials focused on the cardiovascular benefits of new glucose-lowering drugs rather than on the potential benefits of more intensive glycemic therapies. It would be interesting to further explore the effects of these novel hypoglycemic agents on IR levels.

Notably, the present study did not conduct a subgroup analysis according to HF type due to the unavailability of data on left ventricular EF (LVEF). HF was categorized into three subgroups based on LVEF. HF with reduced EF (HFrEF) is defined as an LVEF < 40%, whereas HFpEF is defined as an LVEF > 50%. HF with a mid-range EF (HFmrEF) is identified as an LVEF of 40–49%, which is more similar to HFrEF, especially considering the high prevalence of ischaemic heart disease [49, 50]. Although previous studies investigated the IR indices and clinical outcomes in patients with patients [26, 51], few compared the predictive performance of these IR indices for prognosis in individuals with HFpEF and HFrEF. Sun reported that the TyG index was independently correlated with the risk of major adverse cardiovascular events in patients with HFrEF undergoing percutaneous coronary intervention [52]. Han et al. observed a significant relationship between the TyG index and clinical outcomes in patients with HFrEF, whereas no significant differences were observed in the HFpEF group [43]. However, some studies arrived at different conclusions. Zhou. reported that the TyG index is closely associated with the risk of all-cause death and cardiovascular deaths among patients with HFpEF. The predictive performance of the TyG index is impaired in the HFrEF [26]. Another study concluded that a high TyG index is associated with an increased risk of mortality and rehospitalization in patients with HFpEF [29]. These conflicting results suggest that IR may exert different effects on the different HF phenotypes.

HFpEF is preceded by non-cardiac comorbidities such as hypertension, obesity, and metabolic syndrome, whereas HFrEF is mostly induced by the acute or chronic loss of cardiomyocyte damage due to heart disease [53, 54]. IR, the main feature of metabolic syndrome, is closely associated with extracardiac comorbidities such as diabetes and obesity [26]. A previous study indicated that the prevalence of IR was similar between HF groups [55]; thus, HFrEF and HFpEF may share many common risk factors. These metabolic abnormalities can directly cause cardiovascular stiffening and myocardial fibrosis, resulting in the progression of HFpEF [56, 57].Additionally, patients with IR are more likely to develop cardiac diseases, leading to acute or chronic cardiomyocyte damage and loss. Finally, an imbalance in the heart wall structure is induced, resulting in the development of systolic dysfunction, a hallmark of HErEF [58]. Therefore, the effect of IR may be relatively weak in patients with HFrEF owing to its indirect role. Theoretically, the predictive power of the IR indices was more prominent in the HFpEF versus HFrEF group. These contradictory results may be related to the heterogeneous nature of HFpEF [59]. The interactions between various extracardiac comorbidities may make it challenging to evaluate the effects of IR indices on mortality. Therefore, prospective, randomized studies are required to determine the practical clinical applications of IR in patients with HFpEF and HFrEF.

Although various studies have explored new prognostic markers of HF, the clinical significance of classical prognostic markers should also be also emphasized. NT-proBNP levels reflect HF severity and are significantly associated with adverse outcomes in patients with HF [60]. The marker level decreased in response to the use of standard HF therapies, and increased levels were associated with a poor prognosis. The present study evaluated the latest values of these IR indices and NT-proBNP during hospitalization to examine whether treatment improved them (Supplementary Table 10). According to the results, the NT-proBNP and IR indices decreased significantly after treatment. Furthermore, non-survivors demonstrated significantly higher NT-proBNP level, TyG index, TyG-BMI index, and TG/HDL-C level, suggesting a higher risk of mortality.

This study had some limitations that must be addressed. First, it was a retrospective analysis, meaning that causality could not be definitively established. In addition, there may be residual or unmeasured confounders that could not be addressed. However, we used multifaceted and rigorous statistical methods that consistently yielded similar results, strengthening the validity of our findings. Second, the data were collected from a single center. Owing to the lack of standardization and the absence of reference intervals and cut-offs in the general population and different subgroups, it was difficult to implement the results in clinical practice. These factors should be explored further future studies. Third, glucose and TG levels can change dramatically during hospitalization and do not always reflect a patient’s glycolipid metabolism. Although previous and the present studies confirmed the predictive value of IR indices, it is necessary to explore surrogate markers of IR that represent a steady metabolic status. Fourth, this study failed to report the causes of hospitalization because of the absence of data. Differences in hospitalization causes, which yield different prognoses, could have influenced the results. Finally, the present study focused on critically ill patients and included only patients with CHF who were admitted to the ICU, thereby neglecting those who were ambulatory-treated or admitted to the internal medicine ward. Thus, selection bias may have occurred, leading to difficulty generalizing the results to all patients with CHF.

## Conclusion

To the best of our knowledge, this study is the first to compare the ability of three surrogate indices for IR: the TyG index, the TyG-BMI index, and TG/HDL-C ratio for predicting the 5-year mortality in critically ill patients with CHF. Despite the inclusion of additional risk variables, both the TyG index and TG/HDL-C ratio shown a strong association with 5-year mortality. The relationship between TyG-BMI and 5-year mortality requires further confirmation. Although incorporating these indices into the basic risk model did not result in an improvement in predictive performance for all-cause mortality, the TyG index appears to be the most promising index for prevention and risk stratification in critically ill patients with CHF.

### Electronic supplementary material

Below is the link to the electronic supplementary material.

Supplementary Material 1.

## Data Availability

No datasets were generated or analysed during the current study.

## References

[1] McDonagh TA, Metra M, Adamo M (2021). ESC guidelines for the diagnosis and treatment of acute and chronic heart failure. Eur Heart J.

[2] Jones NR, Roalfe AK, AdokI I (2019). Survival of patients with chronic heart failure in the community: a systematic review and meta-analysis. Eur J Heart Fail.

[3] Valley TS, Sjoding MW, Ryan AM (2017). Intensive care unit admission and survival among older patients with chronic obstructive pulmonary disease, heart failure, or myocardial infarction. Ann Am Thorac Soc.

[4] Shahim B, Kapelios CJ, Savarese G (2023). Global Public Health Burden of Heart Failure: an updated review. Card Fail Rev.

[5] Ziaeian B, Fonarow GC (2016). Epidemiology and aetiology of heart failure. Nat Rev Cardiol..

[6] McDonagh TA, Metra M, Adamo M (2023). Focused update of the 2021 ESC guidelines for the diagnosis and treatment of acute and chronic heart failure. Eur Heart J..

[7] Ho KL, KarwI QG, Connolly D (2022). Metabolic, structural and biochemical changes in diabetes and the development of heart failure. Diabetologia.

[8] Yaribeygi H, Farrokhi FR, Butler AE (2019). Insulin resistance: review of the underlying molecular mechanisms. J Cell Physiol..

[9] Di Pino A, Defronzo RA (2019). Insulin resistance and atherosclerosis: implications for insulin-sensitizing agents. Endocr Rev..

[10] Kim JA, Montagnani M, Koh KK (2006). Reciprocal relationships between insulin resistance and endothelial dysfunction: molecular and pathophysiological mechanisms. Circulation..

[11] Guo W, Zhao L, Mo F (2021). The prognostic value of the triglyceride glucose index in patients with chronic heart failure and type 2 diabetes: a retrospective cohort study. Diabetes Res Clin Pract..

[12] Aroor AR, Mandavia CH, Sowers JR (2012). Insulin resistance and heart failure: molecular mechanisms. Heart Fail Clin..

[13] Zheng L, Li B, Lin S (2019). Role and mechanism of cardiac insulin resistance in occurrence of heart failure caused by myocardial hypertrophy. Aging..

[14] Doehner W, Rauchhaus M, Ponikowski P (2005). Impaired insulin sensitivity as an independent risk factor for mortality in patients with stable chronic heart failure. J Am Coll Cardiol..

[15] Jia G, Hill MA, Sowers JR (2018). Diabetic cardiomyopathy: an update of mechanisms contributing to this clinical entity. Circ Res..

[16] Laakso M, Kuusisto J (2014). Insulin resistance and hyperglycaemia in cardiovascular disease development. Nat Rev Endocrinol..

[17] Li C, Ford ES, McGuire LC (2007). Association of metabolic syndrome and insulin resistance with congestive heart failure: findings from the third National Health and Nutrition Examination Survey. J Epidemiol Community Health..

[18] Muniyappa R, Lee S, Chen H (2008). Current approaches for assessing insulin sensitivity and resistance in vivo: advantages, limitations, and appropriate usage. Am J Physiol Endocrinol Metab..

[19] Zhao X, An X (2023). The crucial role and mechanism of insulin resistance in metabolic disease. Front Endocrinol (Lausanne)..

[20] Guerrero-Romero F, SimentaL-Mendía LE, González-Ortiz M (2010). The product of triglycerides and glucose, a simple measure of insulin sensitivity. Comparison with the euglycemic-hyperinsulinemic clamp. J Clin Endocrinol Metab..

[21] Giannini C, Santoro N (2011). The triglyceride-to-HDL cholesterol ratio: association with insulin resistance in obese youths of different ethnic backgrounds. Diabetes Care..

[22] Ramírez-Vélez R, Pérez-Sousa M, González-Ruíz K (2019). Obesity- and lipid-related parameters in the identification of older adults with a high risk of prediabetes according to the American Diabetes Association: an analysis of the 2015 health, well-being, and aging study. Nutrients.

[23] Shi W, Xing L, Jing L (2020). Value of triglyceride–glucose index for the estimation of ischemic stroke risk: insights from a general population. Nutr Metab Cardiovasc Dis..

[24] Guo W, Zhu W, Wu J (2021). Triglyceride glucose index is associated with arterial stiffness and 10-year cardiovascular disease risk in a Chinese population. Front Cardiovasc Med..

[25] Su WY, Chen SC, Huang YT (2019). Comparison of the effects of fasting glucose, hemoglobin A(1c), and triglyceride–glucose index on cardiovascular events in type 2 diabetes mellitus. Nutrients.

[26] Zhou Y, Wang C, Che H (2023). Association between the triglyceride–glucose index and the risk of mortality among patients with chronic heart failure: results from a retrospective cohort study in China. Cardiovasc Diabetol..

[27] Dou J, Guo C, Wang Y (2023). Association between triglyceride glucose-body mass and 1-year all-cause mortality of patients with heart failure: a retrospective study utilizing the MIMIC-IV database. Cardiovasc Diabetol.

[28] Iwani NA, Jalaludin  MY, Zin RM (2017). Triglyceride to HDL-C ratio is associated with insulin resistance in overweight and obese children. Sci Rep.

[29] Zhou Q, Yang J, Tang H (2023). High triglyceride–glucose (TyG) index is associated with poor prognosis of heart failure with preserved ejection fraction. Cardiovasc Diabetol.

[30] González Ariza A, Navas González FJ, León Jurado J M, et al. Data mining as a tool to infer chicken carcass and meat cut quality from autochthonous genotypes. Animals (Basel), 2022, 12(19) 10.3390/ani12192702 PMC9559234 36230442

[31] Salgado Pardo JI, Navas González FJ, González Ariza A (2023). Study of meat and carcass quality-related traits in Turkey populations through discriminant canonical analysis. Foods.

[32] Xu L, Wu M, Chen S (2022). Triglyceride–glucose index associates with incident heart failure: a cohort study. Diabetes Metab.

[33] Li X, Chan JSK, Guan B (2022). Triglyceride–glucose index and the risk of heart failure: evidence from two large cohorts and a mendelian randomization analysis. Cardiovasc Diabetol..

[34] Zheng H, Chen G, Wu K (2023). Relationship between cumulative exposure to triglyceride–glucose index and heart failure: a prospective cohort study. Cardiovasc Diabetol..

[35] Khalaji A, Behnoush AH, Khanmohammadi S (2023). Triglyceride–glucose index and heart failure: a systematic review and meta-analysis. Cardiovasc Diabetol..

[36] Huang R, Wang Z, Chen J (2022). Prognostic value of triglyceride glucose (TyG) index in patients with acute decompensated heart failure. Cardiovasc Diabetol.

[37] Yang S, Shi X, Liu W (2023). Association between triglyceride glucose-body mass index and heart failure in subjects with diabetes mellitus or prediabetes mellitus: a cross-sectional study. Front Endocrinol (Lausanne).

[38] Butt JH, Petrie MC, Jhund PS (2023). Anthropometric measures and adverse outcomes in heart failure with reduced ejection fraction: revisiting the obesity paradox. Eur Heart J..

[39] Fonarow GC, Srikanthan P, Costanzo MR (2007). An obesity paradox in acute heart failure: analysis of body mass index and inhospital mortality for 108,927 patients in the acute decompensated Heart Failure National Registry. Am Heart J..

[40] Rich JD, Burns J, Freed BH (2018). Meta-analysis global group in chronic (MAGGIC) heart failure risk score: validation of a simple tool for the prediction of morbidity and mortality in heart failure with preserved ejection fraction. J Am Heart Assoc..

[41] Thrainsdottir IS, Aspelund T, Thorgeirsson G (2005). The association between glucose abnormalities and heart failure in the population-based Reykjavik study. Diabetes Care..

[42] Allen LA, Magid DJ, Gurwitz JH (2013). Risk factors for adverse outcomes by left ventricular ejection fraction in a contemporary heart failure population. Circ Heart Fail..

[43] Han S, Wang C, Tong F (2022). Triglyceride glucose index and its combination with the get with the guidelines-heart failure score in predicting the prognosis in patients with heart failure. Front Nutr..

[44] Huang R, Lin Y, Ye X (2022). Triglyceride–glucose index in the development of heart failure and left ventricular dysfunction: analysis of the ARIC study. Eur J Prev Cardiol..

[45] Goode KM, John J, Rigby AS (2009). Elevated glycated haemoglobin is a strong predictor of mortality in patients with left ventricular systolic dysfunction who are not receiving treatment for diabetes mellitus. Heart..

[46] Kristensen SL, Preiss P, Jhund PS (2016). Risk related to pre-diabetes mellitus and diabetes mellitus in heart failure with reduced ejection fraction: insights from prospective comparison of ARNI with ACEI to determine impact on global mortality and morbidity in heart failure trial. Circ Heart Fail.

[47] Suskin N, McKelvie RS, Burns RJ (2000). Glucose and insulin abnormalities relate to functional capacity in patients with congestive heart failure. Eur Heart J..

[48] Hayward RA, Reaven PD, Wiitala WL (2015). Follow-up of glycemic control and cardiovascular outcomes in type 2 diabetes. N Engl J Med..

[49] Vedin O, Lam CSP, Koh AS (2017). Significance of ischemic heart disease in patients with heart failure and preserved, midrange, and reduced ejection fraction: a nationwide cohort study. Circ Heart Fail.

[50] Koh AS, Tay WT, Teng THK (2017). A comprehensive population-based characterization of heart failure with mid-range ejection fraction. Eur J Heart Fail..

[51] Yang Z, Gong H, Kan F (2023). Association between the triglyceride glucose (TyG) index and the risk of acute kidney injury in critically ill patients with heart failure: analysis of the MIMIC-IV database. Cardiovasc Diabetol..

[52] Sun T, Huang X, Zhang B (2023). Prognostic significance of the triglyceride–glucose index for patients with ischemic heart failure after percutaneous coronary intervention. Front Endocrinol (Lausanne)..

[53] Borlaug BA, Melenovsky V, Russell SD (2006). Impaired chronotropic and vasodilator reserves limit exercise capacity in patients with heart failure and a preserved ejection fraction. Circulation..

[54] Packer M (2019). Drugs that ameliorate epicardial adipose tissue inflammation may have discordant effects in heart failure with a preserved ejection fraction as compared with a reduced ejection fraction. J Card Fail.

[55] Son TK, Toan NH, Thang N (2022). Prediabetes and insulin resistance in a population of patients with heart failure and reduced or preserved ejection fraction but without diabetes, overweight or hypertension. Cardiovasc Diabetol..

[56] Sajdeya O, Beran A, Mhanna M (2022). Triglyceride glucose index for the prediction of subclinical atherosclerosis and arterial stiffness: a meta-analysis of 37,780 individuals. Curr Probl Cardiol..

[57] Lau ES, Panah LG, Zern EK (2022). Arterial stiffness and vascular load in HFpEF: differences among women and men. J Card Fail..

[58] Simmonds SJ, Cuijpers I, Heymans S (2020). Cellular and molecular differences between HFpEF and HFrEF: a step ahead in an improved pathological understanding. Cells.

[59] Zheng SL, Chan FT, Nabeebaccus AA (2018). Drug treatment effects on outcomes in heart failure with preserved ejection fraction: a systematic review and meta-analysis. Heart..

[60] Anand IS, Fisher LD, Chiang YT (2003). Changes in brain natriuretic peptide and norepinephrine over time and mortality and morbidity in the valsartan heart failure trial (Val-HeFT). Circulation..

