# Supplementary material for: Comparative study on the predictive value of TG/HDL-C, TyG and TyG-BMI indices for 5-year mortality in critically ill patients with chronic heart failure: a retrospective study
Source: Cardiovasc Diabetol. 2024 Jun 20;23:213. doi: 10.1186/s12933-024-02308-w (PMC11191322; doi:10.1186/s12933-024-02308-w)
Supplement: Supplementary file 1 — Supplementary Material 1. [file 12933_2024_2308_MOESM1_ESM.docx]

**Supplementary Table 1 Association between IR related index and hospital mortality (Cox regression)**

| **Index** | **Groups** | **Non-adjusted**  **HR (95% CI) P-Value** | **Model 1**  **HR (95% CI) P-Value** | **Model 2**  **HR (95% CI) P-Value** |
| --- | --- | --- | --- | --- |
| **TyG** | Continuous | 2.07(1.67-2.58) <0.001 | 1.94(1.53-2.46) <0.001 | 1.63(1.25-2.12) <0.001 |
|  | T1 (≤9.03; N=333) | ref | ref | ref |
|  | T2 (>9.03, ≤9.45; N=333) | 1.6(0.96-2.67) 0.071 | 1.63(0.97-2.72) 0.064 | 1.33(0.79-2.25) 0.281 |
|  | T3(>9.45, ≤9.85; N=333) | 1.56(0.94-2.61) 0.088 | 1.45(0.86-2.43) 0.162 | 1.16(0.68-2) 0.584 |
|  | T4(>9.85; N=330) | 2.79(1.75-4.47) <0.001 | 2.45(1.5-4) <0.001 | 1.7(1.00-2.86) 0.049 |
|  | P for trend | <0.001 | <0.001 | 0.069 |
| **TyG-BMI** | Continuous | 1.004(1.001-1.003) 0.02 | 1.004(1.000-1.003) 0.02 | 1.002(0.99-1.003) 0.2 |
|  | T1 (≤225.06; N=332) | ref | ref | ref |
|  | T2 (>225.06, ≤261.65; N=333) | 0.97(0.6-1.56) 0.895 | 0.99(0.61-1.6) 0.964 | 0.95(0.75-1.22) 0.697 |
|  | T3(>261.65, ≤305.03; N=331) | 1.32(0.84-2.06) 0.226 | 1.4(0.88-2.21) 0.153 | 1.05(0.89-1.23) 0.578 |
|  | T4(>305.03; N=333) | 1.52(0.99-2.35) 0.057 | 1.64(1.04-2.58) 0.035 | 1.06(0.94-1.20) 0.339 |
|  | P for trend | 0.024 | 0.013 | 0.225 |
| **TG/HDL** | Continuous | 1.02(0.99-1.04) 0.142 | 1.01(0.99-1.04) 0.214 | 1.00(0.98-1.03) 0.828 |
|  | T1 (≤1.85; N=333) | ref | ref | ref |
|  | T2 (>1.85, ≤3.11; N=331) | 0.98(0.61-1.56) 0.917 | 0.97(0.61-1.56) 0.908 | 0.94(0.58-1.52) 0.8 |
|  | T3(>3.11, ≤4.88; N=334) | 0.93(0.58-1.5) 0.764 | 1.01(0.63-1.63) 0.96 | 1.02(0.62-1.65) 0.952 |
|  | T4(>4.88; N=331) | 1.78(1.17-2.7) 0.007 | 1.89(1.23-2.9) 0.004 | 1.58(1.01-2.48) 0.044 |
|  | P for trend | 0.006 | 0.003 | 0.03 |

Model 1: Age, AF, Diabetes, CKD, Hypertension, Respiratory failure;

Model 2: Age, AF, Diabetes, CKD, Hypertension, Respiratory failure, ALT, AST, RBC, CK, Creatinine, HbA1c, Hb, NT-proBNP, potassium, sodium, BUN, WBC, ACEI/ARB, Anti-platelet drugs, β-receptor;

**Supplementary Table 2 Association between IR related index and 360-day mortality (Cox regression)**

| **Index** | **Groups** | **Non-adjusted**  **HR (95% CI) P-Value** | **Model 1**  **HR (95% CI) P-Value** | **Model 2**  **HR (95% CI) P-Value** |
| --- | --- | --- | --- | --- |
| **TyG** | Continuous | 1.92(1.6-2.31) <0.001 | 1.9(1.55-2.31) <0.001 | 1.6(1.29-1.99) <0.001 |
|  | T1 (≤9.03; N=333) | ref | ref | ref |
|  | T2 (>9.03, ≤9.45; N=333) | 1.55(1.05-2.3) 0.029 | 1.6(1.08-2.38) 0.020 | 1.32(0.88-1.98) 0.176 |
|  | T3(>9.45, ≤9.85; N=333) | 1.37(0.92-2.06) 0.123 | 1.33(0.88-2) 0.172 | 1.08(0.7-1.65) 0.728 |
|  | T4(>9.85; N=330) | 2.41(1.66-3.48) <0.001 | 2.28(1.54-3.36) <0.001 | 1.62(1.07-2.46) 0.023 |
|  | P for trend | <0.001 | <0.001 | 0.05 |
| **TyG-BMI** | Continuous | 1.002(1.000-1.003) 0.05 | 1.002(1.000-1.004) 0.026 | 1.002(1.00-1.004) 0.12 |
|  | T1 (≤225.06; N=332) | ref | ref | ref |
|  | T2 (>225.06, ≤261.65; N=333) | 1.07(0.74-1.57) 0.711 | 1.11(0.76-1.63) 0.581 | 1.05(0.72-1.55) 0.789 |
|  | T3(>261.65, ≤305.03; N=331) | 1.34(0.94-1.93) 0.111 | 1.46(1.01-2.11) 0.046 | 1.29(0.88-1.9) 0.197 |
|  | T4(>305.03; N=333) | 1.42(1.00-2.03) 0.053 | 1.56(1.07-2.27) 0.020 | 1.36(0.91-2.03) 0.133 |
|  | P for trend | 0.025 | 0.008 | 0.083 |
| **TG/HDL** | Continuous | 1.02(1.01-1.04) 0.009 | 1.02(1.00-1.04) 0.01 | 1.01(0.99-1.03) 0.178 |
|  | T1 (≤1.85; N=333) | ref | ref | ref |
|  | T2 (>1.85, ≤3.11; N=331) | 0.97(0.66-1.41) 0.864 | 0.97(0.67-1.42) 0.882 | 0.91(0.62-1.34) 0.64 |
|  | T3(>3.11, ≤4.88; N=334) | 0.97(0.67-1.41) 0.877 | 1.03(0.7-1.5) 0.896 | 0.99(0.67-1.45) 0.954 |
|  | T4(>4.88; N=331) | 1.65(1.18-2.32) 0.004 | 1.77(1.25-2.51) 0.001 | 1.53(1.06-2.19) 0.022 |
|  | P for trend | 0.003 | 0.001 | 0.014 |

Model 1: Age, AF, Diabetes, CKD, Hypertension, Respiratory failure;

Model 2: Age, AF, Diabetes, CKD, Hypertension, Respiratory failure, ALT, AST, RBC, CK, Creatinine, HbA1c, Hb, NT-proBNP, potassium, sodium, BUN, WBC, ACEI/ARB, Anti-platelet drugs, β-receptor;

**Supplementary Table 3 HR and p values for all of the variable in the model 2**

**(TyG index Cox regression)**

| **Index** | **Model 2**  **HR (95% CI) P-Value** | |
| --- | --- | --- |
| Age | 1.02 (1.01~1.03) | <0.001 |
| AF | 1.2 (0.96~1.52) | 0.113 |
| Diabetes | 1.19 (0.92~1.54) | 0.189 |
| CKD | 0.99 (0.74~1.32) | 0.929 |
| Hypertension | 0.83 (0.63~1.09) | 0.183 |
| Respiratory failure | 1.38 (1.25~2.01) | <0.001 |
| ALT | 1 (1~1) | 0.345 |
| AST | 1 (1~1) | 0.051 |
| RBC | 1.02 (0.74~1.42) | 0.886 |
| CK | 1 (1~1) | 0.02 |
| Creatinine | 0.98 (0.91~1.06) | 0.702 |
| HbA1c | 1.05 (0.99~1.13) | 0.12 |
| Hb | 0.92 (0.82~1.03) | 0.162 |
| NT-proBNP | 1 (1~1) | 0.122 |
| Potassium | 1.06 (0.94~1.19) | 0.336 |
| Sodium | 1 (0.97~1.02) | 0.691 |
| BUN | 1.01 (1~1.01) | <0.001 |
| WBC | 1.02 (1.01~1.03) | <0.001 |
| ACEI/ARB | 0.72 (0.53~0.97) | 0.032 |
| Anti-platelet drugs | 0.81 (0.65~1.01) | 0.06 |
| β-receptor | 0.78 (0.62~0.98) | 0.032 |

Model II: Age, AF, Diabetes, CKD, Hypertension, Respiratory failure, ALT, AST, RBC, CK, Creatinine, HbA1c, Hb, NT-proBNP, potassium, sodium, BUN, WBC, ACEI/ARB, Anti-platelet drugs, β-receptor;

**Supplementary Table 4 HR and p values for all of the variable in the model 2**

**(TyG-BMI index Cox regression)**

| **Index** | **Model 2**  **HR (95% CI) P-Value** | |
| --- | --- | --- |
| Age | 1.02 (1.01~1.04) | <0.001 |
| AF | 1.15 (0.91~1.45) | 0.235 |
| Diabetes | 1.22 (0.95~1.58) | 0.122 |
| CKD | 0.95 (0.71~1.26) | 0.708 |
| Hypertension | 0.81 (0.62~1.07) | 0.132 |
| Respiratory failure | 1.64 (1.29~2.08) | <0.001 |
| ALT | 1 (1~1) | 0.471 |
| AST | 1 (1~1) | 0.071 |
| RBC | 0.97 (0.7~1.35) | 0.874 |
| CK | 1 (1~1) | 0.023 |
| Creatinine | 0.98 (0.91~1.06) | 0.682 |
| HbA1c | 1.06 (0.99~1.13) | 0.096 |
| Hb | 0.93 (0.83~1.05) | 0.237 |
| NT-proBNP | 1 (1~1) | 0.073 |
| Potassium | 1.08 (0.96~1.22) | 0.197 |
| Sodium | 0.99 (0.97~1.02) | 0.517 |
| BUN | 1.01 (1~1.01) | <0.001 |
| WBC | 1.02 (1.01~1.02) | <0.001 |
| ACEI/ARB | 0.73 (0.54~0.99) | 0.042 |
| Anti-platelet drugs | 0.78 (0.62~0.98) | 0.029 |
| β-receptor | 0.77 (0.61~0.96) | 0.022 |

Model II: Age, AF, Diabetes, CKD, Hypertension, Respiratory failure, ALT, AST, RBC, CK, Creatinine, HbA1c, Hb, NT-proBNP, potassium, sodium, BUN, WBC, ACEI/ARB, Anti-platelet drugs, β-receptor;

**Supplementary Table 5 HR and p values for all of the variable in the model 2 (TG/HDL-C index Cox regression)**

| **Index** | **Model 2**  **HR (95% CI) P-Value** | |
| --- | --- | --- |
| Age | 1.02 (1.01~1.03) | <0.001 |
| AF | 1.19 (0.95~1.5) | 0.132 |
| Diabetes | 1.23 (0.95~1.58) | 0.114 |
| CKD | 0.94 (0.7~1.25) | 0.658 |
| Hypertension | 0.83 (0.63~1.1) | 0.192 |
| Respiratory failure | 1.62 (1.28~2.06) | <0.001 |
| ALT | 1 (1~1) | 0.509 |
| AST | 1 (1~1) | 0.125 |
| RBC | 1 (0.72~1.39) | 0.992 |
| CK | 1 (1~1) | 0.033 |
| Creatinine | 0.99 (0.91~1.07) | 0.755 |
| HbA1c | 1.06 (1~1.14) | 0.062 |
| Hb | 0.93 (0.83~1.04) | 0.214 |
| NT-proBNP | 1 (1~1) | 0.094 |
| Potassium | 1.09 (0.97~1.23) | 0.167 |
| Sodium | 1 (0.97~1.02) | 0.68 |
| BUN | 1.01 (1~1.01) | <0.001 |
| WBC | 1.02 (1.01~1.03) | <0.001 |
| ACEI/ARB | 0.73 (0.54~0.99) | 0.046 |
| Anti-platelet drugs | 0.8 (0.64~1) | 0.055 |
| β-receptor | 0.79 (0.63~0.99) | 0.039 |

Model II: Age, AF, Diabetes, CKD, Hypertension, Respiratory failure, ALT, AST, RBC, CK, Creatinine, HbA1c, Hb, NT-proBNP, potassium, sodium, BUN, WBC, ACEI/ARB, Anti-platelet drugs, β-receptor;

**Supplementary Table 6** Discrimination of each predictive model for outcomes

| **Models** | **AUC (95% CI)** | **Cut-off** | **Sensitivity** | **Specificity** | **P-Value** |
| --- | --- | --- | --- | --- | --- |
| **5-year mortality** |  |  |  |  |  |
| TyG index | 0.608(0.571-0.645) | 10.15 | 0.28 | 0.94 | ref |
| TyG-BMI index | 0.558(0.522-0.594) | 266.72 | 0.56 | 0.57 | 0.012 |
| TG/HDL-C | 0.561(0.524-0.598) | 5.3 | 0.31 | 0.83 | 0.007 |
| **360-day mortality** |  |  |  |  |  |
| TyG index | 0.607(0.566-0.647) | 10.15 | 0.27 | 0.92 | ref |
| TyG-BMI index | 0.547(0.508-0.587) | 272.23 | 0.51 | 0.59 | 0.007 |
| TG/HDL-C | 0.556(0.514-0.597) | 5.3 | 0.32 | 0.81 | 0.009 |
| **Hospital mortality** |  |  |  |  |  |
| TyG index | 0.624(0.575-0.673) | 10.15 | 0.31 | 0.91 | ref |
| TyG-BMI index | 0.559(0.512-0.607) | 272.23 | 0.54 | 0.59 | 0.017 |
| TG/HDL-C | 0.554(0.503-0.605) | 5.10 | 0.35 | 0.79 | 0.003 |

**Supplementary Table 7** **Association between TyG index and mortality**

| **Models** | **Non-adjusted**  **HR (95% CI) P-Value** | **Model 1**  **HR (95% CI) P-Value** | **Model 2**  **HR (95% CI) P-Value** |
| --- | --- | --- | --- |
| **5-year mortality** |  |  |  |
| <10.15 | ref | ref | ref |
| ≥10.15 | 3.58(2.82-4.56) <0.001 | 3.44(2.66-4.46) <0.001 | 2.79(2.22-3.68) <0.001 |
| **360-day mortality** |  |  |  |
| <10.15 | ref | ref | ref |
| ≥10.15 | 3.2(2.42-4.24) <0.001 | 3.28(2.43-4.42) <0.001 | 2.61(1.89-3.62) <0.001 |
| **Hospital mortality** |  |  |  |
| <10.15 | ref | ref | ref |
| ≥10.15 | 3.33(2.36-4.7) <0.001 | 3.33(2.31-4.8) <0.001 | 2.71(1.82-4.04) <0.001 |

**Supplementary Table 8 Association between continuous TyG levels and 5-year mortality in subgroups**

| **Subgroups** | **Model 2**  **HR (95% CI) P-Value** | | ***P* for interaction** |
| --- | --- | --- | --- |
| **Age** |  |  | 0.982 |
| ≤70 | 1.89(1.42-2.52) | 0.001 |  |
| >70 | 1.90(1.56-2.32) | 0.001 |  |
| **AF** |  |  | 0.871 |
| No | 2.05(1.62-2.6) | 0.001 |  |
| Yes | 1.98(1.58-2.48) | 0.001 |  |
| **Diabetes** |  |  | 0.243 |
| No | 1.99(1.63-2.42) | 0.001 |  |
| Yes | 1.67(1.23-2.26) | 0.001 |  |
| **CKD** |  |  |  |
| No | 1.76(1.38-2.24) | 0.001 | 0.586 |
| Yes | 1.96(1.57-2.45) | 0.001 |  |
| **Hypertension** |  |  |  |
| No | 1.94(1.62-2.33) | 0.001 | 0.595 |
| Yes | 1.73(1.23-2.45) | 0.002 |  |
| **Respiration failure** |  |  | 1.00 |
| No | 1.77(1.39-2.27) | 0.001 |  |
| Yes | 1.71(1.37-2.13) | 0.001 |  |
| **CK** |  |  | 0.219 |
| ≤198 | 2.2(1.71-2.83) | 0.001 |  |
| >198 | 1.76(1.43-2.17) | 0.001 |  |
| **HbA1c** |  |  | 0.532 |
| ≤6 | 1.65(1.15-2.37) | 0.006 |  |
| >6 | 1.92(1.58-2.32) | 0.001 |  |
| **NT-proBNP** |  |  | 0.321 |
| ≤6700 | 2.1(1.62-2.72) | 0.012 |  |
| >6700 | 1.85(1.51-2.27) | 0.001 |  |

**Supplementary Table 9** Improvement in discrimination for mortality after adding TyG indices

| **Models** | **AUC (95% CI)** | **P value*** |
| --- | --- | --- |
| **5-year mortality** |  |  |
| Basic model* | 0.722 (0.697-0.746) |  |
| +TyG index | 0.746 (0.721-0.769) | 0.0051 |
| **360-day mortality** |  |  |
| Basic model* | 0.736 (0.711-0.759) |  |
| +TyG index | 0.760 (0.736-0.782) | 0.0111 |
| **Hospital mortality** |  |  |
| Basic model* | 0.749 (0.725-0.772) |  |
| +TyG index | 0.776 (0.752-0.798) | 0.0271 |

Basic model*: Age, Sex, Systolic blood pressure, BMI, Diabetes, Hypertension, Respiratory failure, AF, AMI, Old-AMI, Dyslipidemia, ACEI/ARB, Anti-platelet drug, PCI, Statin, Anti-coagulant drug, β-receptor, Diuretics, NT-proBNP, Troponin-I, and CK.

**Supplementary Table 10 The last value of NT-proBNP and IR indices before charge**

| **Variables** | | **Total**  **(n = 1329) p value**^#^ | **Survivor**  **(n = 997) p value** | **Non-survivor**  **(n = 332) p value** | **p** |
| --- | --- | --- | --- | --- | --- |
| Before | NT-proBNP | 12139.9 ± 14064.0 | 11038.2 ± 13669.5 | 15448.0 ± 14720.2 | < 0.001 |
|  | TyG | 9.5 ± 0.6 | 9.4 ± 0.6 | 9.7 ± 0.8 | < 0.001 |
|  | TyG-BMI | 270.0 ± 66.68 | 267.1 ± 66.4 | 278.6 ± 66.7 | 0.002 |
|  | TG/HDL-C | 4.2 ± 4.8 | 3.9 ± 4.5 | 4.9 ± 5.5 | 0.006 |
| After | NT-proBNP | 9182.7 ± 11987.4 (<0.001) ^#^ | 8061.7 ± 11475.9 (0.007) ^#^ | 12549.2 ± 12848.6 (<0.001) ^#^ | < 0.001 |
|  | TyG | 8.7 ± 0.6 (<0.001) ^#^ | 8.7 ± 0.5 (<0.001) ^#^ | 8.9 ± 0.6 (<0.001) ^#^ | < 0.001 |
|  | TyG-BMI | 249.2 ± 62.0 (<0.001) ^#^ | 247.2± 62.6 (<0.001) ^#^ | 255.1 ± 59.7 (<0.001) ^#^ | 0.046 |
|  | TG/HDL-C | 3.0 ± 2.5 (<0.001) ^#^ | 2.8 ± 2.3 (<0.001) ^#^ | 3.4 ± 2.9 (<0.001) ^#^ | < 0.001 |

**p value**^#^: p value for before and after treatment (The last test value of variable)
